# Targeting NRF2 for the Treatment of Friedreich’s Ataxia: A Comparison among Drugs

**DOI:** 10.3390/ijms20205211

**Published:** 2019-10-21

**Authors:** Sara Petrillo, Jessica D’Amico, Piergiorgio La Rosa, Enrico Silvio Bertini, Fiorella Piemonte

**Affiliations:** Unit of Muscular and Neurodegenerative Diseases, Bambino Gesù Children’s Hospital, IRCCS, 00146 Rome, Italy; sara.petrillo@opbg.net (S.P.); jessica.damico@opbg.net (J.D.); piergiorgio.larosa@opbg.net (P.L.R.); enricosilvio.bertini@opbg.net (E.S.B.)

**Keywords:** Friedreich’s Ataxia, Nrf2, redox active drugs, neurodegenerative disease, frataxin

## Abstract

NRF2 (Nuclear factor Erythroid 2-related Factor 2) signaling is impaired in Friedreich’s Ataxia (FRDA), an autosomal recessive disease characterized by progressive nervous system damage and degeneration of nerve fibers in the spinal cord and peripheral nerves. The loss of frataxin in patients results in iron sulfur cluster deficiency and iron accumulation in the mitochondria, making FRDA a fatal and debilitating condition. There are no currently approved therapies for the treatment of FRDA and molecules able to activate NRF2 have the potential to induce clinical benefits in patients. In this study, we compared the efficacy of six redox-active drugs, some already adopted in clinical trials, targeting NRF2 activation and frataxin expression in fibroblasts obtained from skin biopsies of FRDA patients. All of these drugs consistently increased NRF2 expression, but differential profiles of NRF2 downstream genes were activated. The Sulforaphane and *N*-acetylcysteine were particularly effective on genes involved in preventing inflammation and maintaining glutathione homeostasis, the dimethyl fumarate, omaxevolone, and EPI-743 in counteracting toxic products accumulation, the idebenone in mitochondrial protection. This study may contribute to develop synergic therapies, based on a combination of treatment molecules.

## 1. Introduction

The activation of the transcription factor NRF2 (Nuclear factor Erythroid 2-related Factor 2) counteracts many of the pathological processes occurring in neurodegenerative diseases [1,2,3]. Thus, targeting NRF2 signaling may provide a therapeutic option to delay onset, slow progression, and ameliorate symptoms of several neurodegenerative disorders, whose pathogenesis is mediated by oxidative stress [1,2,3,4].

NRF2 regulates many cytoprotective pathways through the activation of antioxidant defenses, inhibition of inflammation, improvement of mitochondrial function, and maintenance of protein homeostasis [1,5], thus it is an attractive pharmacologic target for neuroprotection in chronic diseases [2,3,6].

The impairment of NRF2 is becoming an hallmark in Friedreich’s Ataxia (FRDA), a severe neurodegenerative disease for which no cure or effective treatments are available so far [7,8,9,10,11,12]. 

In FRDA, an intronic GAA repeat of the FXN gene results in the deficiency of the frataxin (fxn) protein, causing iron sulfur cluster (ISC) assembly defects, mitochondrial iron accumulation, and impairment of the antioxidant defense [9,13,14,15,16,17].

Moving from our previous data [8,11] and from several lines of evidence overall showing decreased NRF2 activity either in FXN knockdown cells and in multiple FRDA models [7,9,12], in this study we investigate the effectiveness of several compounds, some of which are already adopted in clinical trials [18,19,20,21,22,23,24], to induce the NRF2 pathway and their impact on frataxin expression. Indeed, NRF2-inducible ARE sequences are present in the promoter of the frataxin gene and are completely conserved across evolution [10], thus we wanted to verify a comparative FXN expression up-regulation with several NRF2 inducers. For this purpose, we treated in vitro cultured fibroblasts of FRDA patients with redox-active compounds, some of those currently used for the treatment of neurodegenerative and mitochondrial diseases.

Four classes of compounds have been selected: (1) those directly acting on Nrf2, by releasing it from its specific inhibitor Keap-1 (sulforaphane, SFN, and dimethyl fumarate, DMF) [18,19,20]; (2) those involved in mitochondrial function (idebenone and EPI-743) [25]; (3) one classical antioxidant (*N*-acetylcysteine, NAC), actively involved in redox cellular balance, but whose action on NRF2 system has not yet been established and (4) the cyclic cyanoenone RTA 408 (OMAVeloxolone, OMAV), a new Nrf2 activator that prevents the ubiquitination of Nrf2 [26].

Many of those compounds are already adopted in clinical trials, such as DMF that is used for the treatment of relapsing multiple sclerosis (Tecfidera, Biogen-Idec) [18,19,20], EPI-743 evaluated in mitochondrial diseases [25,27,28], idebenone, in treating Leber hereditary optic neuropathy (LHON) [29,30] and in FRDA [31,32], and NAC showing protective effects in frataxin-deficient cell types and in ethylmalonic encephalopathy [33,34].

Exploring the NRF2 pathway in FRDA and its modulation by “old” and “new” compounds may help to better understand the FRDA pathogenesis and contribute to develop innovative therapies, also taking into account the emerging “mechanism-based” approach to diseases that was recently proposed by Cuadrado et al. [2].

## 2. Results

### 2.1. The Pathway of NRF2 Is Down-Regulated in Fibroblasts of FRDA Patients

**Figure 1:**
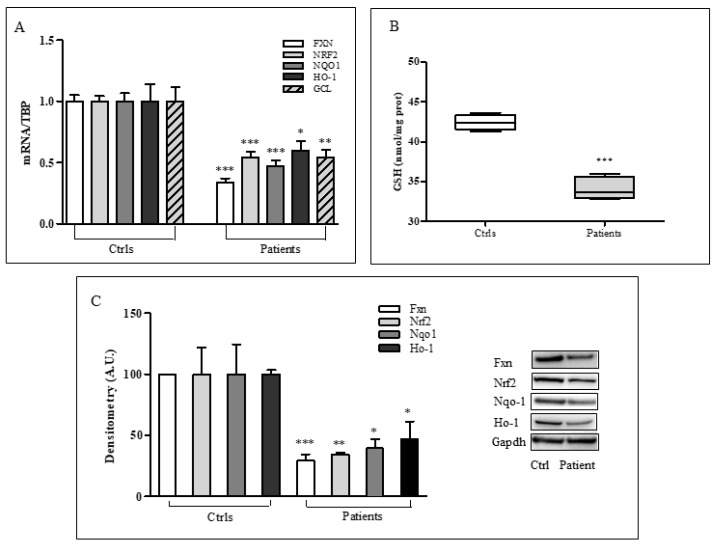
NRF2 and downstream genes expression in fibroblasts of patients with Friedreich’s Ataxia (FRDA). (**A**) Real-time PCR analysis of frataxin (FXN) and NRF2-target genes (NQO1, HO-1, GCL) in fibroblasts obtained from skin biopsies of three patients with FRDA. (**B**) Glutathione (GSH) content in FRDA fibroblasts. (**C**) Representative western blot (**right**) and densitometric analysis (**left**) of fxn, Nqo1, and Ho-1 protein levels. Experiments were conducted in triplicates and values expressed as mean ± SEM. * *p* < 0.05, ** *p* < 0.01, *** *p* < 0.001, compared with controls’ group (Ctrls).

Quantitative PCR and western blot analyses were performed to measure NRF2 expression in fibroblasts obtained from skin biopsies of three patients with FRDA (Figure 1). NRF2 function is partially impaired, with a 71% frataxin decrease leading to a 46% defect in mRNA NRF2 (Figure 1A) and a 60% reduction of protein level (Figure 1C). Similarly, NRF2 main target genes, NAD(P)H: quinone oxidoreductase 1 (NQO1), Heme Oxygenase-1 (HO-1) and γ-glutamylcysteine ligase (GCL), are reduced in patients’ cells, either as mRNA (53% NQO1, 40% HO-1, 46% GCL, Figure 1A) and as proteins’ amount (60% Nqo1, 53% Ho-1, Figure 1C). According to GCL transcripts, even the glutathione (GSH) content appears significantly reduced (20%) in FRDA fibroblasts with respect to controls’ cells (34 ± 0.7 vs. 42 ± 0.4 nmol/mg prot., Figure 1B), further confirming previous evidence of imbalanced glutathione homeostasis in this disease [9,12,35,36,37,38,39].

### 2.2. The NRF2 Expression Is Modulated by Redox-Active Drugs

**Figure 2:**
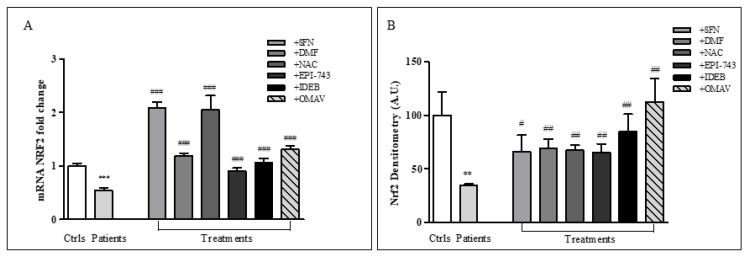
Drugs-mediated NRF2 induction in FRDA fibroblasts. (**A**) NRF2 mRNA and (**B**) densitometry of Nrf2 protein amount after 24 h treatments with 10 µM SFN, 30 µM DMF, 100 µM NAC, 1 µM EPI-743, 1 µM IDEB, 100 nM OMAV. TBP has been used for normalization. Relative quantification of gene expression was performed according to the 2^−ΔΔ*C*t^ method. Values represent mean ± SEM of three independent experiments ** *p* < 0.01, *** *p* < 0.001, compared with controls’ group (Ctrls); ^#^ *p* < 0.05, ^##^ *p* < 0.01, ^###^ *p* < 0.001, respect to untreated patients.

Given the growing role of NRF2 as therapeutic target in FRDA and in several chronic diseases [2,3], we evaluated the effect of six redox compounds on NRF2 expression in order to design a potential drugs classification. We treated FRDA fibroblasts with SFN, DMF, NAC, EPI-743, Idebenone, and OMAV for 24 h and analyzed the NRF2 gene (Figure 2A) and protein amount (Figure 2B). All drugs significantly increased NRF2 transcripts at 24 h treatments, with SFN and NAC that were particularly active (3.9-fold increase SFN and 3.8-fold increase NAC, Figure 2A), compared to untreated cells. DMF, EPI-743, Idebenone and OMAV, although were less active than SFN and NAC, however consistently induced NRF2 expression, leading patients’ values close to controls’ level (Figure 2A). A similar trend was observed by western blot analysis, with significant increases of Nrf2 protein amount following all drug treatments (Figure 2B).

### 2.3. Redox-Active Drugs Promote Differential Patterns of NRF2 Induction in FRDA Fibroblasts

**Figure 3:**
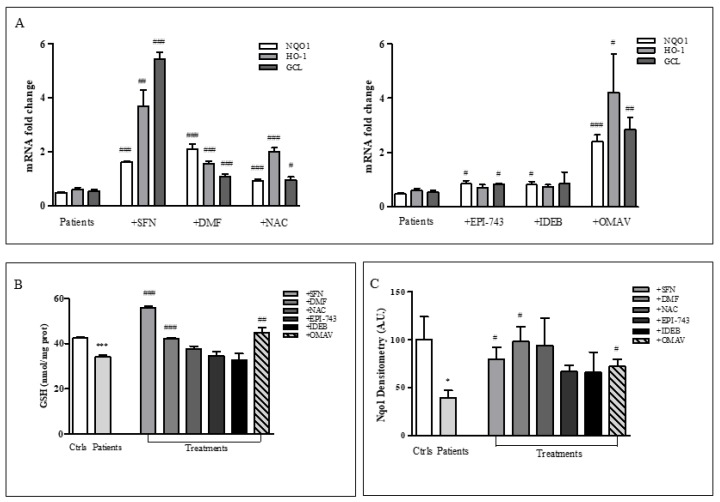
Differential patterns of drugs-mediated NRF2 induction. (**A**) Real-time PCR analysis of NRF2 target genes following SFN, DMF, NAC, EPI-743, IDEB, and OMAV treatments. TBP was used for normalization. Relative quantification of gene expression was performed according to the 2^−ΔΔ*C*t^ method. (**B**) GSH content and (**C**) Nqo1 protein amount after treating FRDA fibroblasts for 24 h with 10 µM SFN, 30 µM DMF, 100 µM NAC, 1 µM EPI-743, 1 µM IDEB, and 100 nM OMAV. Values represent mean ± SEM of three independent experiments. * *p* < 0.05, *** *p* < 0.001, compared with controls’ group (Ctrls); ^#^ *p* < 0.05, ^##^ *p* < 0.01, ^###^ *p* < 0.001, compared to untreated patients.

As NRF2 deficiency negatively affects its down-stream genes (NQO1, HO-1, GCL, Figure 1) and drugs activate NRF2 at different extent (Figure 2), we verified that NRF2 target genes could also be differently regulated by redox drugs, driving differential responses (Figure 3). Thus, we analyzed the molecular profiles of NQO1, HO-1 and GCL after treatments with SFN, DMF, NAC, EPI-743, Idebenone and OMAV (Figure 3A). As expected by the extent of NRF2 activation (Figure 2A), SFN strongly increased the expression of all genes analyzed, particularly GCL and HO-1 that showed 10.1-fold and 6.1-fold increases, respectively, when compared to untreated FRDA cells. Also DMF significantly increased the expression of NRF2 down-stream genes, although more addressed towards the NQO1 induction (4.5-fold increase). Surprisingly NAC, despite representing the substrate supplier for GCL, appears to direct its action mainly towards the activation of HO-1 (3.4-fold increase), compared to NQO1 (2.0-fold increase) and GCL (1.7-fold increase). OMAV is highly efficient on all the NRF2 target genes tested, with a preferential induction for NQO1 (5.1-fold increase), whereas EPI-743 and Idebenone, although pushing towards the NRF2 target genes induction, exhibited a lesser extent of activation than other drugs. These drug-driven gene profiles reflect on the GSH content (Figure 3B) and on Nqo1 protein amount (Figure 3C), which increased especially following SFN, DMF, and OMAV treatments.

### 2.4. NRF2 Inducers Increase the Expression of FXN Gene in FRDA Fibroblasts

**Figure 4:**
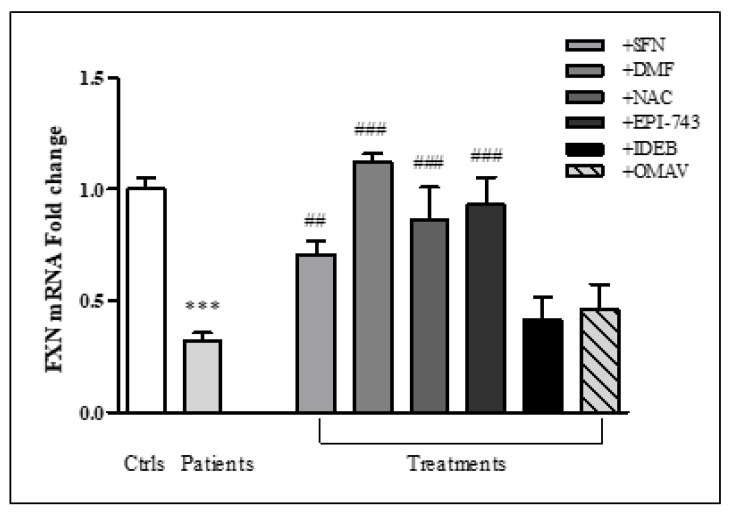
The FXN expression increases in FRDA fibroblasts following the drugs-mediated NRF2 induction. Real-time PCR analysis of FXN after 24 h treatment with 10 µM SFN, 30 µM DMF, 100 µM NAC, 1 µM EPI-743, 1 µM IDEB, 100 nM OMAV. TBP has been used for normalization. Relative quantification of FXN gene expression was performed according to the 2^−ΔΔ*C*t^ method. Values represent mean ± SEM of three independent experiments. *** *p* < 0.001, compared with controls’ group (Ctrls); ^##^ *p* < 0.01, ^###^ *p* < 0.001, respect to untreated patients.

Besides the positive effect of drugs on NRF2 induction, the hallmark in FRDA remains the fxn deficiency. Thus we explored whether, and to what extent, drugs were able to increase the FXN expression in FRDA fibroblasts, while also considering the important evidence provided by Sahdeo et al. [10] showing the existence of evolutionarily conserved NRF2-binding sites (AREs) in the FXN gene. As reported in Figure 4, SFN, DMF, NAC, and EPI-743 significantly increased FXN transcripts (2.2-fold SFN, 3.5-fold DMF, 2.7-fold NAC, 2.9-fold EPI-743), while OMAV and Idebenone caused only a modest increase of the FXN gene expression, not reaching statistical significance. Importantly, SFN, DMF, NAC, and EPI-743 caused an FXN increase of more than 2 times with respect to the untreated cells, thus leading patients’ FXN levels to become close to those of asymptomatic carriers (ranging 40–50% of normal) [40]. Of note, our results on DMF-mediated increase of FXN gene expression confirms the recent paper by Jasoliya et al. [41] showing a significant FXN gene induction in vitro and in vivo FRDA cells treated with DMF.

### 2.5. The Drug-Mediated NRF2 Induction Is Time Dependent

**Figure 5:**
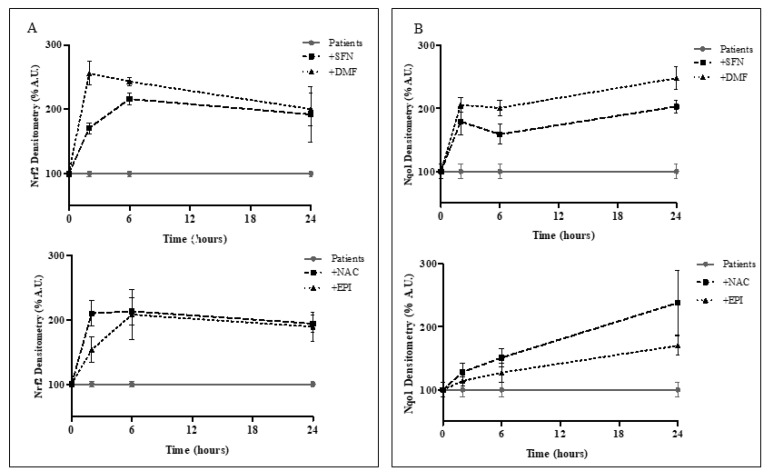
Time-course of drugs-mediated Nrf2 induction. (**A**) Nrf2 and (**B**) Nqo1 protein levels in FRDA fibroblasts, as quantified by Western Blot analyses after 2, 6, 24 h treatment with 10 µM SFN, 30 µM DMF (**up**) and 100 µM NAC, 1 µM EPI-743 (**down**). Experiments were performed in triplicates and values expressed as % densitometry respect to untreated patients.

All drugs induce a fast (2 h) Nrf2 increase, which grows up to 6 h until stabilizing after 24 h treatment (Figure 5A). Only DMF reaches its maximum of activation at 2 h and then slowly decreases after 24 h drug administration. The Nqo1 expression displays a similar trend (Figure 5B), with a first fast enhancement at 2 h treatment for SFN and DMF and a progressive increase over time for NAC and EPI-743 up to 24 h. The ability of Nrf2 to “auto-modulate” its expression may explain the Nrf2 stabilization at longer time of drugs exposition, thus ensuring a constant level of Nrf2 expression and a correct redox balance in cells.

### 2.6. The NRF2 Deficiency is Mediated by “Keap-1/DJ-1/p62 Axis” in FRDA Fibroblasts

**Figure 6:**
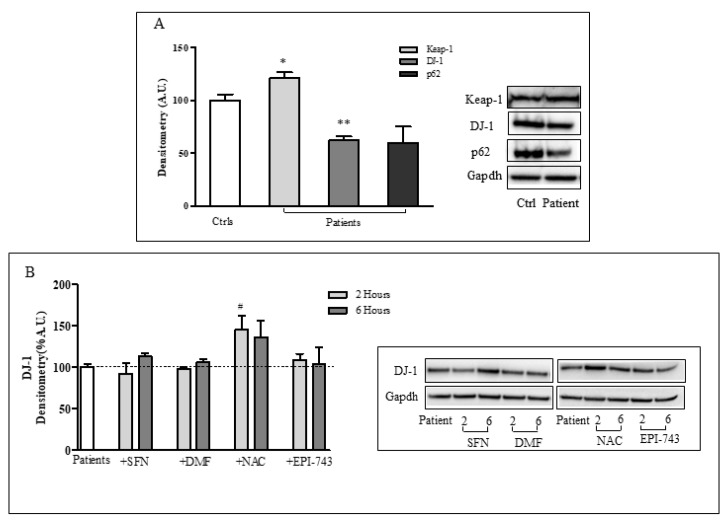
A defective “Keap-1/DJ-1/p62 axis” underlies Nrf2 deficiency in FRDA fibroblasts. (**A**) Representative western blot (right) and densitometric analysis (left) of Keap-1, DJ-1 and p62 proteins in FRDA fibroblasts. (**B**) Representative western blot (**right**) and densitometric analysis (**left**) of DJ-1 protein level after 2 and 6 h treatments of FRDA fibroblasts with 10 µM SFN, 30 µM DMF, 100 µM NAC, 1 µM EPI-743. Glyceraldehyde-3-phosphate dehydrogenase (Gapdh) was used as a loading control. Experiments were conducted in triplicates and values expressed as mean ± SEM. * *p* < 0.05, ** *p* < 0.01, compared with controls’ group (Ctrls); ^#^ *p* < 0.05, respect to untreated patients. The dotted line represents the expression of DJ1 in patients (considered as 100% expression)

To go deeper into the mechanism underlying the NRF2 down-regulation in FRDA, we further analyzed the expression of the Nrf2 specific inhibitor Kelch-like ECH-associated protein 1 (Keap-1). In particular, we evaluated if the decrease of Nrf2 was a consequence of the Keap-1 stabilization or may depend on its reduced degradation [12]. Our findings show that the expression of Keap-1 was significantly increased (21%) in FRDA fibroblasts (Figure 6A), thus supporting the study by Anzovino et al. [12] showing Keap1 up-regulation in the fxn-deficient heart of a mouse cardiac model of FRDA. Then, we analyzed the expression of p62, the protein responsible for Keap-1 degradation [42], and DJ-1 that acts as a stabilizer of Nrf2 by preventing the Keap1/Nrf2 association [43]. Both DJ-1 and p62 proteins were decreased in FRDA fibroblasts (38% and 40% reduced levels, respectively), but only DJ-1 reached statistical significance (Figure 6A). Furthermore, as DJ-1 is an important redox-sensitive protein known to be inhibited by the formation of a disulfide bond with the oxidized GSH [44], we analyzed the effect of redox drugs on DJ-1 protein levels. As shown in Figure 6B, among Nrf2 inducers, only NAC significantly increased the amount of DJ-1, displaying a 45% enhancement level after 2 h of treatment. The short response time of NAC leads us to assume a post-translational mechanism of DJ-1 regulation in FRDA, with the protein partially inhibited by oxidation in basal conditions and NAC acting as a reducing agent to recover the protein function. The oxidative modulation of DJ-1 has been already described in a model of dopaminergic neuronal (Neuro2A) cells [44], with important implications in the pathogenesis of Parkinson’s Disease.

Overall our findings, besides confirming the impairment of NRF2 in FRDA and evidencing a role for the “Keap-1/DJ1/p62” axis in mediating its deficiency, highlight the susceptibility of the transcription factor to the drugs’ induction, and provide suggestions for a “multi-target” drugs’ effect of the NRF2-mediated protection.

## 3. Discussion

This study moves from the pathogenic hypothesis underlying FRDA according to which the NRF2 dysfunction, as a consequence of fxn deficiency, leads to decreased mitochondrial antioxidant protection, increased reactive oxygen species, and neurodegeneration [7,8,9,11,12,45]. NRF2 activation is neuroprotective in models of neurological disorders such as Parkinson’s disease and multiple sclerosis [6,46], thus, the activation of NRF2 signaling may also be an attractive pharmacological target for neuroprotection in FRDA.

Nrf2 represents an endogenous stabilizer of cell homeostasis, depending on its multifaceted cytoprotective roles as coordinator of many transcriptional networks [3,47]. Indeed, NRF2 affects multiple pathways, including oxidative, inflammatory, and metabolic alterations [2], thus identifying NRF2 as a central therapeutic target may be critical, especially in the new perspective of “the NRF2 diseasome”, a systems medicine approach where NRF2 alterations represent a common mechanism in a network of diseases [2,3].

Defects in the pathway of NRF2 have been described in several in vitro and in vivo models of FRDA. In Knock-in Knock-out (KIKO) and GAA repeat expansion YG8R FRDA mice, NRF2 deficiency causes mitochondrial impairment and oxidative imbalance [48,49], such as in frataxin-silenced motor neurons and in human fibroblasts, where defective NRF2 nuclear translocation and down regulation of NRF2 target genes have been reported [7,8,9,11].

Because of the versatile role of NRF2 and its high reactivity towards many electrophilic xenobiotics, many NRF2 inducers have been described and new agents continue to be discovered [2,50]. However, due to this multi-target benefit, the NRF2 modulation could be considered “not specific”, which would weaken its importance as a valuable drug target, in spite of its central role in several chronic diseases [2,3]. Thus, understanding if drug specific profiles are activated in response to NRF2 induction might be very useful to target therapies.

Starting from this assumption and from evidence showing the efficacy of NRF2 induction on FRDA models [11,21,36,48,49], in this study we compared the expression profile of NRF2 and its downstream genes after the administration of six different drugs known to modulate NRF2. Many of the selected compounds have been already adopted in clinical trials [18,19,20,21,22,23,24]. DMF, for instance, is used for the treatment of multiple sclerosis and was recently proposed for clinical validation in PD [24]. OMAV has undergone clinical trials in non-small cell lung cancer and in a clinical trial of FRDA (ClinicalTrials.gov registration number: NCT02255435). EPI-743 is currently under evaluation in mitochondrial diseases [21]; (https://clinicaltrials.gov/ct2/show/NCT02352896; https://clinicaltrials.gov/ct2/show/NCT0164205) and many clinical trials have been performed with idebenone in FRDA [21]. Also NAC has been used in clinical practice for many years, although the mechanisms underlying its clinical application still remain unclear [22].

A number of drugs used in this study have been currently analyzed for their efficacy on NRF2 induction on in vitro FRDA models [10,11,36,51]. The OMAV-mediated NRF2 induction, for instance, displayed beneficial effects on cerebellar granule neurons of KIKO FRDA mouse and in human fibroblasts [49]; SFN was efficient in counteracting the hypersensitivity to oxidation and reducing lipid peroxidation in FRDA fibroblasts of mouse models [48]; SFN, NAC, and EPI-743 showed NRF2-mediated neuro-protective effects in frataxin-silenced motor neurons and in neural stem cells isolated from KIKO mice [11,36,51].

In this study, we show that all drugs consistently increased NRF2 expression in fibroblasts of patients with FRDA but, interestingly, differential patterns of activation were induced. SFN was particularly effective on GCL and HO-1, whereas DMF and OMAV significantly increased NQO1, and NAC addressed its action mainly towards HO-1.

Important functional implications arise from these distinct drug-mediated activation profiles: Nqo1, by catalyzing the reduction of quinone to hydroquinone, will prevent the toxic accumulation of quinone products and counteract lipid peroxidation [52]; Ho-1, playing a protective role in the early phases of immune response, will contribute to modulate the inflammation [53,54]; Gcl, which is responsible for the de novo synthesis of glutathione, will be essential in buffering the redox tissue imbalance [55,56,57]. Therefore, our findings, besides confirming a central role for NRF2 in the pathogenesis of FRDA, can contribute to developing targeted therapies, through the use of drugs aimed at preventing inflammation (i.e., SFN, NAC), lipid peroxidation (DMF, OMAV, EPI-743), or redox imbalance (SFN), based on the patient’s clinical conditions and their therapeutic needs.

We are aware that fibroblasts are not the major tissue affected in FRDA, however they recapitulate many molecular features of the disease, including the reduced frataxin expression and the NRF2 impairment, thus representing a manageable resource for drug screening [49,58].

Drugs elicit different mechanisms of actions and NRF2 is regulated at multiple levels [59,60], thus we believe that the specific expression profiles evidenced in this study may depend on the multi-target control underlying NRF2 modulation. SFN, for instance, displays a dual mechanism: it directly reacts with Cys residues on Keap1, leading to the release and nuclear translocation of NRF2 [5,61], but it also functions by a Keap1-independent mechanism due to Nrf2 phosphorylation, inhibition of GSK3β-dependent Fyn, and Akt modulation [62,63]. Similarly, DMF has multiple functions either acting on Keap1 thiols or also inhibiting GSK3β [64,65]. Unlike SFN and DMF, OMAV displays a more specific activation of NRF2, through the direct inhibition of Keap1 on its primary sensor C^151^, which prevents the Nrf2 ubiquitination [66]. A multifaceted activity has been described even for NAC [22,67], whereas the NRF2 induction mediated by idebenone and EPI-743 is not still deeply elucidated. Physiologically, idebenone acts as an electron carrier within the mitochondrial respiratory chain [23]. It displays similar antioxidant properties as its structural analogue CoQ_10_, but the decreased molecular weight and the increased water solubility make idebenone more bioavailable than CoQ_10_ [68,69]. EPI-743 has been recently demonstrated preventing in vitro ferroptosis by the specific inhibition of 15-lipoxygenase enzyme activity [70]. It was effective on a neuronal model of FRDA and in mitochondrial diseases [27,28,36,51,71].

However, we must keep in mind that the molecular hallmark in FRDA is the low expression of functional frataxin protein and therapeutic efforts have to be focused on attempting to increase FXN mRNA and/or protein amount [72].

FRDA carriers remain asymptomatic up to approximately 50% protein level, thus a small increase of FXN expression can become curative. Importantly, ARE sequences have been identified in the FXN locus, upstream the transcription start site of FXN and are completely conserved across evolution [10]. Thus, approaches based on the NRF2 modulation may expand the therapeutic opportunities for this disease. Among drugs tested in this study, SFN, DMF, NAC, and EPI-743 consistently elevated the FXN gene expression in FRDA fibroblasts. Importantly, the FXN gene was increased by three times in patients’ cells, respecting the untreated FRDA cells and thus leading to pathologic FXN levels close to carriers’ range.

Then, we attempted to go deeper in the mechanism underlying the NRF2 impairment in FRDA. Thus, also moving from the study by Anzovino et al. [12] that reported a dysfunctional “Nrf2-Keap1 axis” in the heart of a FXN KO mouse model, we analyzed the expression of Keap-1 and two proteins (DJ-1 and p62) responsible for “Keap1-mediated” Nrf2 stabilization in FRDA fibroblasts [42,43]. Confirming Anzovino’s findings, we found an up-regulation of Keap-1 in FRDA fibroblasts, and additionally, we found significant decreases of DJ-1 and p62 proteins amount, indicative of a reduction of Nrf2 stability in the disease. Of note, the NAC treatment displayed a short-term effect on DJ-1, thus suggesting a mechanism of DJ-1 regulation mediated by reversible oxidation [44].

Overall, this study strengthens the role of NRF2 as a central therapeutic target in FRDA and contributes to position FRDA in the “NRF2 diseasome”, a new approach assembling disease phenotypes joined by NRF2 defects [2,3]. Furthermore, the identification of differential molecular profiles in FRDA will be useful for designing innovative therapies that are also based on a drugs combination.

## 4. Materials and Methods

### 4.1. Fibroblasts Cultures

Skin biopsies were taken from three clinically affected (and genetically proven) FRDA patients (two males and one female) (Table 1) and three age-matched controls (Ctrls). Fibroblasts were grown in Dulbecco’s modified Eagle’s medium supplemented with 10% fetal bovine serum, 50 units/mL penicillin, 50 µg/mL streptomycin, 0.4% (*v*/*v*), at 37 °C in 5% CO_2_. Fibroblasts were cultured to 70% confluence and incubated for 2, 6, and 24 h with 10 µM SFN, 30 µM DMF, 100 µM NAC, 1 µM EPI-743, 1 µM Idebenone, and 100 nM OMAV diluted in culture medium (drugs dosing has been chosen following [11,36,73]). After washing, cells were lysed in Total RNA Purification Plus Kit (Norgen Biotek Corp., Torold, ON, Canada), according to the manufacturer’s protocol for RNA extraction and subjected to quantitative Real-Time PCR, or lysed with RIPA buffer including DTT and protease inhibitors for Western blotting analysis. Cells were used at similar 9–11 passage numbers and were tested for mycoplasma contamination. The assays were performed in triplicates. All the participants signed an informed consent and the study was approved by the Ethics Committee of “Bambino Gesù” Children’s Hospital (code 1166/2016; date of approval 08/06/2016).

### 4.2. Quantitative Real-Time PCR (qRT-PCR)

1 µg RNA samples was reverse transcribed with the SuperScript™ First-Strand Synthesis system and random hexamers as primers (Life Technologies, Carlsbad, CA, USA). The expression levels of FXN, NRF2, NQO1, HO-1, and GCL were measured by qRT-PCR in an ABI PRISM 7500 Sequence Detection System (Life Technologies) using Power SYBR Green I dye chemistry (ThermoFisher Scientific, Walthman, MA, USA). Data were analyzed using the 2^−∆∆*C*t^ method with TBP (TATA box binding protein) and Glyceraldehyde-3-phosphate dehydrogenase (GAPDH) as housekeeping genes, and data are shown as fold change relative to controls. Primers used for qRT-PCR are reported in Petrillo et al. [74].

### 4.3. GSH Assay

Glutathione (GSH) levels were detected in fibroblasts using the enzymatic re-cycling assay [75], with minor modifications. Briefly, samples were resuspended in dH_2_O and sonicated 2 times for 2 sec, de-proteinized with 5% (*w*/*v*) sulphosalycilic acid (SSA, Sigma-Aldrich, St. Louis, MO, USA) and the glutathione content was determined after dilution of the acid-soluble fraction in Na-phosphate buffer containing EDTA (pH 7.5). GSH concentrations were measured with the ThioStar^®^ glutathione detection reagent (Arbor Assays, Michigan, MI, USA), using GSH as standard (Sigma Chemicals, St. Louis, MO, USA). The fluorescence was measured by an EnSpire^®^ Multimode Plate Reader (Perkin Elmer, Waltham, MA, USA). Protein concentration (mg·mL^−1^) was detected by the BCA method (ThermoFisher, Walthman, MA, USA) and GSH levels were expressed as nmol/mg prot.

### 4.4. Western Blot Analysis

Fibroblasts (1 × 10^6^) were lysed on ice with RIPA buffer, including DTT and protease inhibitors. 40 µg proteins were subjected to SDS PAGE on 4–12% denaturing gel and probed with the following antibodies: Nrf2 (1:500, Abcam, Cambridge, UK), Nqo1 (1:7000, Novus Biologicals, Minneapolis, MN, USA), Ho-1 (1:3000, Abcam), Frataxin (1:500, Santa Cruz Biotechnology, Dallas, TX, USA), and GAPDH (1:10,000, Sigma Aldrich) as loading control. Immunoreactive bands were detected using the Lite Ablot Extend Long Lasting Chemiluminescent substrate (Euroclone, Milan, Italy). Signals derived from appropriate HRP-conjugated secondary antibodies (Bethyl Laboratories, Montgomery, TX, USA) were captured by Chemi DocTM XRS 2015 (Bio-Rad Laboratories, Hercules, CA, USA) and densitometric analysis was performed using Image Lab software (Version 5.2.1, Bio-Rad Laboratories)

### 4.5. Statistical Analysis

Statistical analysis was performed using the GRAPHPAD/Prism 5.0 Software (San Diego, CA, USA). Statistically significant differences between groups were analyzed using Student’s *t*-test for normally distributed variables. All data are presented as mean ± standard error. Statistical significance was defined as * *p* < 0.05, ** *p* < 0.001, *** *p* < 0.001 compared to healthy controls, and ^#^ *p* < 0.05, ^##^ *p* < 0.01, ^###^ *p* < 0.001 compared to untreated cells.

## 5. Conclusions

Different redox drugs, joined by their ability to modulate the NRF2 pathway, elicit differential response profiles in FRDA fibroblasts. Using drugs aimed at preventing inflammation (SFN, NAC), lipid peroxidation (DMF, OMAV, and EPI-743), or redox imbalance (SFN), “multi-target” synergic therapies can be developed in FRDA, based on the patient’s clinical conditions and therapeutic needs.

## Figures and Tables

**Table 1:** Clinical data of patients with FRDA.

Patient	Age (Yrs)	Sex	GAA Repeats	Cardiomyopathy	Diabetes
#1	19	F	680/350	No	No
#2	14	M	848/848	Yes	No
#3	8	M	448/848	Yes	No
